# Eliminating acetate formation improves citramalate production by metabolically engineered *Escherichia coli*

**DOI:** 10.1186/s12934-017-0729-2

**Published:** 2017-06-21

**Authors:** Naga Sirisha Parimi, Ian A. Durie, Xianghao Wu, Afaq M. M. Niyas, Mark A. Eiteman

**Affiliations:** School of Chemical, Materials and Biomedical Engineering, Driftmier Engineering Center, University of Georgia, Athens, GA 30602 USA

**Keywords:** Acetyl-CoA, Pyruvate, Citramalate, *Escherichia coli*, Acetate, Glucose, Fed-batch

## Abstract

**Background:**

Citramalate, a chemical precursor to the industrially important methacrylic acid (MAA), can be synthesized using *Escherichia coli* overexpressing citramalate synthase (*cimA* gene). Deletion of *gltA* encoding citrate synthase and *leuC* encoding 3-isopropylmalate dehydratase were critical to achieving high citramalate yields. Acetate is an undesirable by-product potentially formed from pyruvate and acetyl-CoA, the precursors of citramalate during aerobic growth of *E. coli*. This study investigated strategies to minimize acetate and maximize citramalate production in *E. coli* mutants expressing the *cimA* gene.

**Results:**

Key knockouts that minimized acetate formation included acetate kinase (*ackA*), phosphotransacetylase (*pta*), and in particular pyruvate oxidase (*poxB*). Deletion of glucose 6-phosphate dehydrogenase (*zwf*) and ATP synthase (*atpFH*) aimed at improving glycolytic flux negatively impacted cell growth and citramalate accumulation in shake flasks. In a repetitive fed-batch process, *E. coli gltA leuC ackA*-*pta poxB* overexpressing *cimA* generated 54.1 g/L citramalate with a yield of 0.64 g/g glucose (78% of theoretical maximum yield), and only 1.4 g/L acetate in 87 h.

**Conclusions:**

This study identified the gene deletions critical to reducing acetate accumulation during aerobic growth and citramalate production in metabolically engineered *E. coli* strains. The citramalate yield and final titer relative to acetate at the end of the fed-batch process are the highest reported to date (a mass ratio of citramalate to acetate of nearly 40) without being detrimental to citramalate productivity, significantly improving a potential process for the production of this five-carbon chemical.

## Background

Synthetic biology and metabolic engineering have enabled sustainable and eco-friendly manufacturing of commercially important food products, pharmaceuticals, commodity chemicals, and other high value products using microorganisms. Some chemicals which cannot be synthesized exclusively by a biosynthetic route might be generated using hybrid approaches involving both biological and chemical synthesis. For example, methacrylic acid (MAA), a commodity chemical with an estimated annual global market of 2.2 million tons [42] is a monomer of poly(methyl methacrylate) or PMMA, which is used in automobile, construction, medical device, lighting and the home appliance industries. Acrylates in general are very toxic [3], and their direct microbial synthesis at relevant concentrations seems unlikely. MAA can fortunately be synthesized via a hybrid route: biochemical production of citramalate from glucose [40], and subsequently transforming this compound chemically using base-catalyzed decarboxylation and dehydration [22]. Citramalate (or citramalic acid) is naturally found in the metabolic pathways of some anaerobic bacteria [8, 18, 32].

**Fig. 1:**
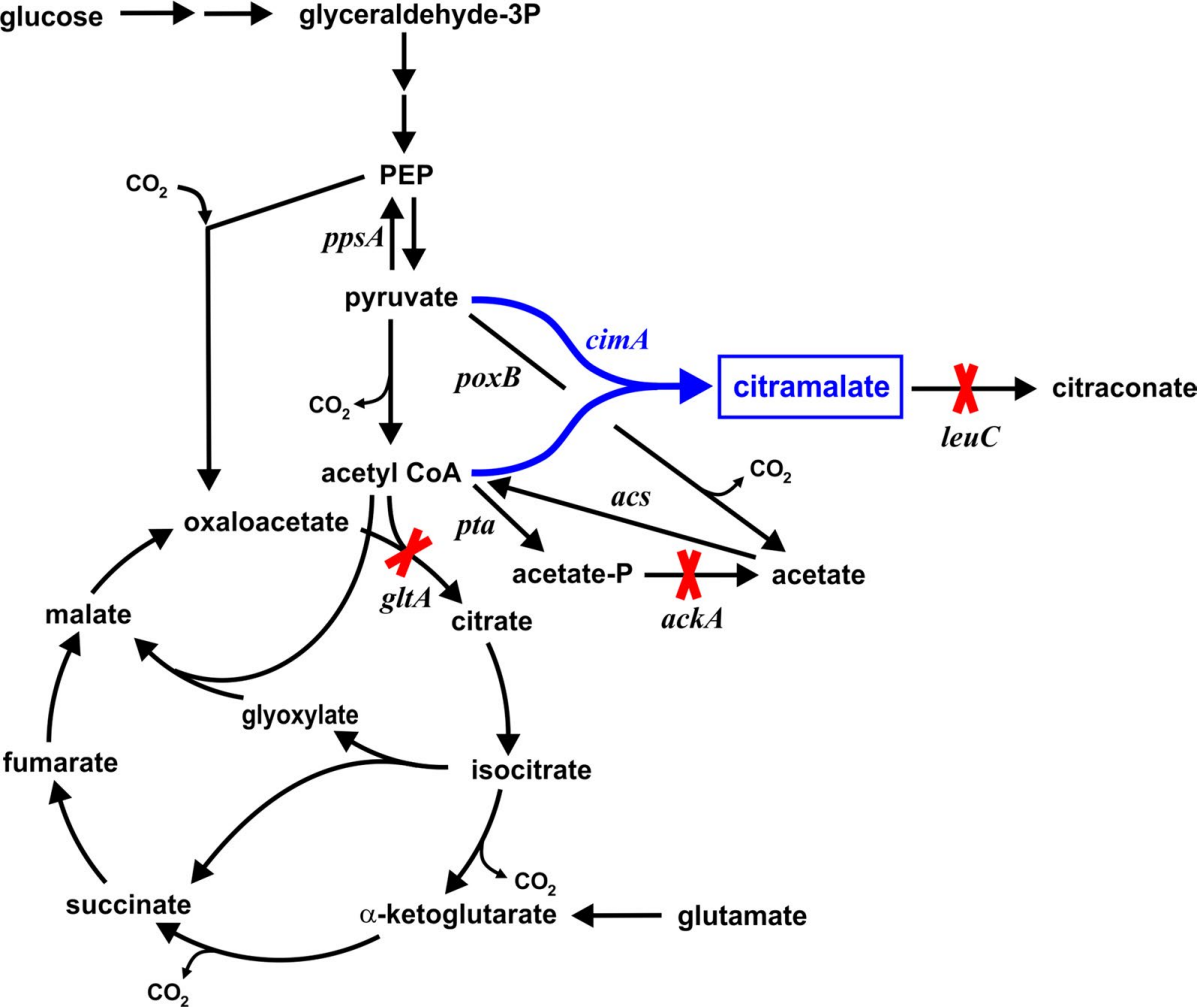
Metabolic pathways for citramalate synthesis in *Escherichia coli* expressing *cimA* coding citramalate synthase (*blue*). Key enzymes (and corresponding genes) are: citrate synthase (*gltA*), 3-isopropylmalate dehydratase (*leuC*), acetate kinase (*ackA*), phosphotransacetylase (*pta*), acetyl-CoA synthetase (*acs*), pyruvate oxidase (*poxB*), phosphoenolpyruvate synthase (*ppsA*). All strains examined in this study had deletions in the *gltA*, *leuC* and *ackA* genes as indicated in *red*

*Escherichia coli* is a well-established microbial cell factory for the biotechnology industry. Citramalate production in metabolically engineered *E. coli* expressing citramalate synthase coding the *cimA* gene (Fig. 1) has previously been demonstrated [4, 40]. In a recent study, *E. coli* MG1655 *gltA leuC ackA/*pZE12-*cimA* containing three key knockouts (citrate synthase, 3-isopropylmalate dehydratase and acetate kinase) accumulated 46 g/L citramalate from glucose at a yield of 0.63 g/g (75% of the theoretical maximum). However, 10 g/L acetate also accumulated despite the deletion of acetate kinase. At the end of the fermentation, this by-product accumulated to an ultimate mass ratio citramalate-to-acetate (i.e., desirable product:undesirable product) of less than 5. Acetate is a typical “overflow” metabolite when wild-type *E. coli* cells are grown at a high growth rate, and the carbon flux into central metabolic pathways exceeds the cells’ biosynthetic demands and the capacity for energy generation [2, 17]. Acetate formation is undesirable because this acid: (i) negatively impacts cell growth even at concentrations as low as 0.5 g/L [28]; (ii) is a sink which diverts carbon that could otherwise be used to synthesize the desired product [17]; (iii) necessitates additional downstream separation step(s) that add to process costs. Acetate is generated by two pathways in *E. coli*: from acetyl-CoA via acetate kinase and phosphotransacetylase (*ackA* and *pta* genes), and from pyruvate via pyruvate oxidase (*poxB*). While the *ackA*-*pta* pathway is typically the route during exponential growth, pyruvate oxidase becomes active during late exponential and early stationary phases [15]. Deletion of *ackA* and/or *pta* genes has previously resulted in lower growth rates and lower but still significant acetate accumulation in several *E. coli* mutants [9, 11, 14, 15]. Strains with *poxB* deleted but not the *ackA*-*pta* pathway accumulated either similar or slightly lower acetate compared to wild type strains [15, 25]. Growth of strains in which both acetate producing pathways were deleted showed very low acetate accumulation and growth rates similar to wild type strains [9, 15].

The two precursors for citramalate, pyruvate and acetyl-CoA, are generated through the glycolytic pathway, and increasing the flux through glycolysis might improve citramalate productivity and yield. Since the ATP/ADP ratio controls glycolysis [24], previous research has shown that decreasing ATP generation increased the rate of glycolysis [30], and product formation [34, 45]. Glycolytic flux may also be improved by preventing flux through the pentose phosphate pathway (PP pathway), for example, by a deletion in glucose 6-phosphate dehydrogenase (*zwf*) [43].

The goal of this study was to improve the formation of citramalate in *E. coli* expressing citramalate synthase by blocking acetate formation. We also investigated whether strategies to increase glycolytic flux would increase citramalate yield and productivity.

## Results

### Citramalate and acetate formation in shake flasks

**Fig. 2:**
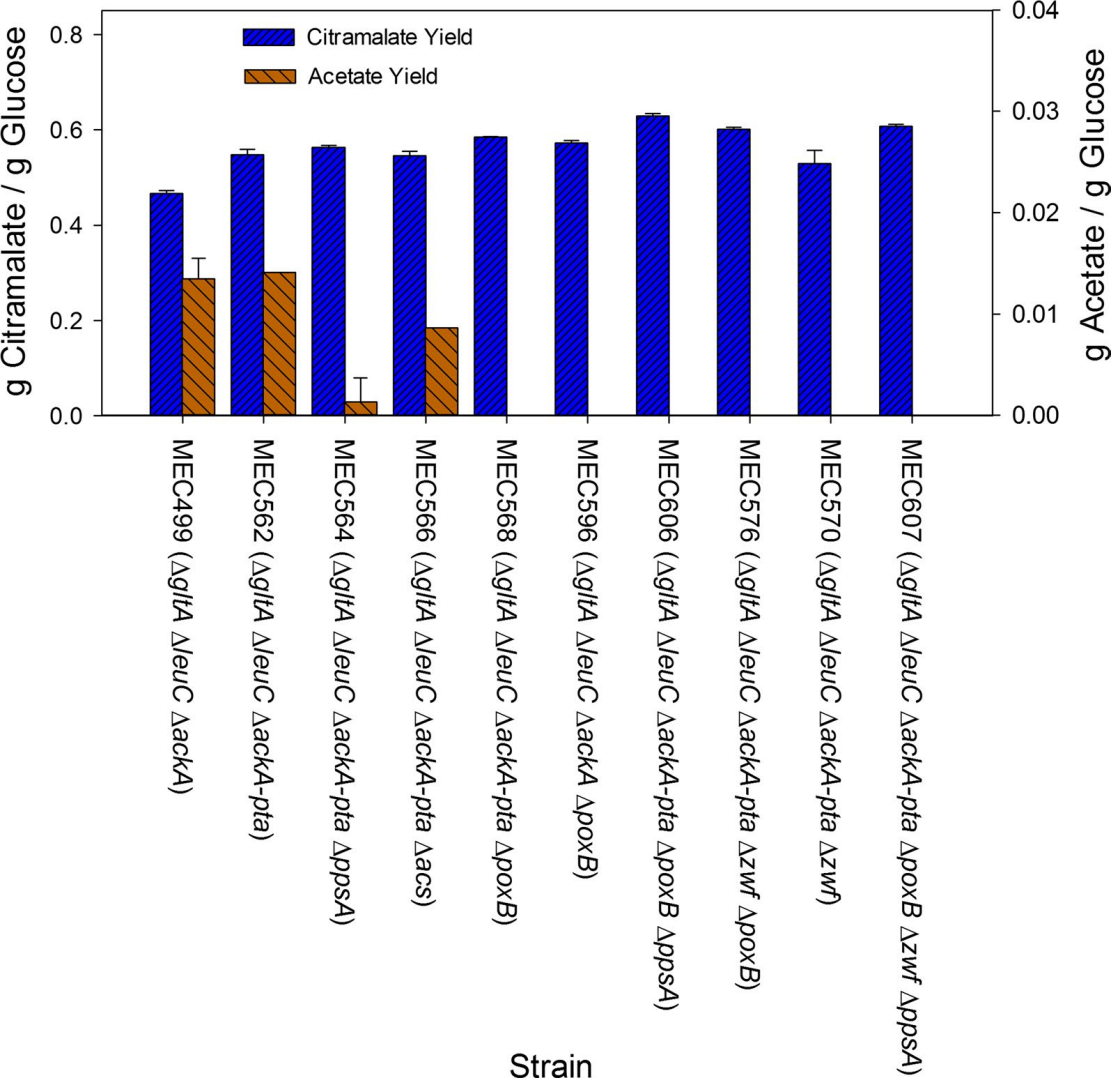
Comparison of citramalate and acetate yields at 24 h in shake flasks using various *E. coli* strains each expressing *cimA* gene on the pZE12-*cimA* plasmid. The defined medium contained 5 g/L glucose, 1 g/L l-glutamate and 0.2 g/L l-leucine. All studies were carried out in triplicate. Results with MEC499/pZE12-*cimA* are from [40]

Citramalate synthase (coded by the *cimA* gene) mediates the conversion of pyruvate and acetyl-CoA to citramalate. Knockouts in the *gltA*, *leuC* and *ackA* genes coding for citrate synthase, 3-isopropylmalate dehydratase, and acetate kinase, respectively, were critical to achieving high citramalate yield [40]. Despite the deletion of acetate kinase, over 10 g/L acetate accumulated in a repetitive fed-batch process [40]. We therefore compared citramalate formation after 24 h in shake flasks by several *E. coli* strains having additional gene knockouts and expressing the pZE12-*cimA* plasmid (Fig. 2). MEC499/pZE12-*cimA* (*gltA leuC ackA*) serves as the control strain [40]. Specifically, we examined the enzymes involved in acetate formation from acetyl-CoA and pyruvate, the precursors of citramalate. We anticipated that screening at the 50 mL shake flask scale in shake flasks would provide guidance on strains to study in greater detail at the 1 L scale in a bioreactor.

In *E. coli* phosphotransacetylase (*pta* gene) and acetate kinase (*ackA*) normally produce acetate during the exponential growth phase through the high energy acetyl phosphate (acetyl-P) intermediate [33]. Previous research demonstrated that acetyl-P can form acetate even in the absence of *ackA* [39]. Since an *ackA* deletion alone in MEC499 was previously shown to be insufficient to prevent acetate formation [40], we suspected acetyl-P generated via phosphotransacetylase might be responsible for acetate formation. From 5.0 g/L glucose, MEC562/pZE12-*cimA* (*gltA leuC ackA*-*pta*) attained an OD of 2.70 and accumulated 2.72 g/L citramalate and 0.07 g/L acetate, similar to the amount of these products observed previously in shake flasks using MG1655 *gltA leuC ackA* [40]. Although the combination of *pta* and *ackA* did not eliminate acetate formation, MEC562/pZE12-*cimA* did show a significant increase in citramalate yield compared to MEC499/pZE12-*cimA* (Fig. 2).

Phosphoenolpyruvate synthase (*ppsA*) catalyzes the ATP-dependent conversion of pyruvate to phosphoenolpyruvate [6]. A loss of pyruvate through this enzyme could affect citramalate accumulation. However, MEC564/pZE12-*cimA* (*gltA leuC ackA*-*pta ppsA*) generated 2.76 g/L citramalate and 0.01 g/L acetate. A knockout of phosphoenolpyruvate synthase did not impact citramalate yield compared to MEC562/pZE12-*cimA*, though surprisingly this knockout reduced acetate formation in shake flasks (Fig. 2).

Acetyl-CoA synthetase (*acs*) is described as an acetate scavenging enzyme that typically converts acetate to acetyl-CoA [7]. To rule out possible reverse formation of acetate via this enzyme, we constructed MEC566/pZE12-*cimA* (*gltA leuC ackA*-*pta acs*) containing the additional knockout in *acs* gene. MEC566/pZE12-*cimA* generated 1.9 g/L citramalate (0.55 g/g yield) and 0.03 g/L acetate. Since the three knockouts *ackA*-*pta acs* did not eliminate acetate formation (Fig. 2), acetate is likely derived from another metabolite and not acetyl CoA. Moreover, the OD was 30% lower for MEC566/pZE12-*cimA*, and not all glucose was consumed at 24 h compared to MEC562/pZE12-*cimA*.

Membrane-bound pyruvate oxidase (*poxB*) is coupled to the respiratory chain, and oxidizes pyruvate directly to acetate, by-passing acetyl-CoA formation. MEC568/pZE12-*cimA* (*gltA leuC ackA*-*pta poxB*) generated 2.9 g/L citramalate, and no acetate was detected. To address whether pyruvate oxidase or phosphotransacetylase was the more important route to acetate formation, we also examined the performance of the strain retaining the native phosphotransacetylase activity. MEC596/pZE12-*cimA* (*gltA leuC ackA poxB*) generated 2.41 g/L citramalate, and no acetate was detected. These results suggest that pyruvate oxidase plays the more important role in acetate formation. MEC596/pZE12-*cimA* also had 20% lower 24 h OD compared to MEC568/pZE12-*cimA*. MEC606/pZE12-*cimA* (*gltA leuC ackA*-*pta poxB ppsA*) accumulated only 2.14 g/L citramalate but no acetate, and grew to an OD of 1.92, nearly 30% lower than MEC562/pZE12-*cimA*. MEC568/pZE12-*cimA* showed a significantly increased citramalate yield (0.58 g/g) compared to MEC562/pZE12-*cimA*, while MEC596/pZE12-*cimA* showed modest increase in citramalate yield (0.57 g/g) compared to MEC562/pZE12-*cimA* (p = 0.06). In summary, the combination of *pta* and *poxB* knockouts appears to be most effective in providing high citramalate yield and preventing acetate formation (Fig. 2). Of course, shake flask results do not necessarily scale to results observed under prolonged conditions in a controlled bioreactor, necessitating additional studies.

Several other strains were examined which were anticipated to benefit citramalate formation, though not affect acetate generation directly. Glucose-6P dehydrogenase (*zwf*) diverts metabolic flux at glucose-6P from glycolysis into the pentose phosphate pathway, which not only reduces glycolytic flux, but also lowers the yield of pyruvate and acetyl-CoA [43]. To examine the impact of this pathway on citramalate formation, three strains containing the *zwf* gene deletion were constructed. Each of these strains having the additional *zwf* deletion showed slightly lower citramalate yields (Fig. 2). However, because they consistently grew much slower than the corresponding strain containing the *zwf* gene, the final citramalate concentrations were much lower (1.31–1.74 g/L).

Previous results have demonstrated that lowering the cellular ATP level increases glycolytic flux [24, 30], increases ethanol yield in yeast [34], and increases pyruvate yield in recombinant *E. coli* [45]. However, MEC638/pZE12-*cimA* (*gltA leuC ackA*-*pta poxB atpFH*) was unable to grow in the glucose/glutamate/leucine defined medium.

In summary, the *poxB* knockout was shown to be important in reducing acetate accumulation in a strain having *gltA leuC ackA* knockouts and expressing citramalate synthase. The *zwf* knockout significantly reduced growth rate, while the *atpFH* knockout prevented growth altogether.

### Citramalate and acetate formation in bioreactors

Results from screening strains in shake flasks do not necessarily transfer to a bioreactor which unavoidably operates under different environmental conditions (mixing, oxygenation, pH control, etc.). We therefore selected a few strains based on encouraging shake flask results for studies at the larger scale. The *poxB* knockout appeared important for the elimination of acetate, while several other single or combinations of gene deletions severely reduced growth. To confirm the importance of *poxB* and more carefully observe differences between key gene knockouts, we selected for controlled batch studies the experimental control strain MEC499/pZE12-*cimA* (*gltA leuC ackA*), and also MEC562/pZE12-*cimA* (*gltA leuC ackA*-*pta*), MEC568/pZE12-*cimA* (*gltA leuC ackA*-*pta poxB*), MEC596/pZE12-*cimA* (*gltA leuC ackA poxB*) and MEC606/pZE12-*cimA* (*gltA leuC ackA*-*pta poxB ppsA*).

**Fig. 3:**
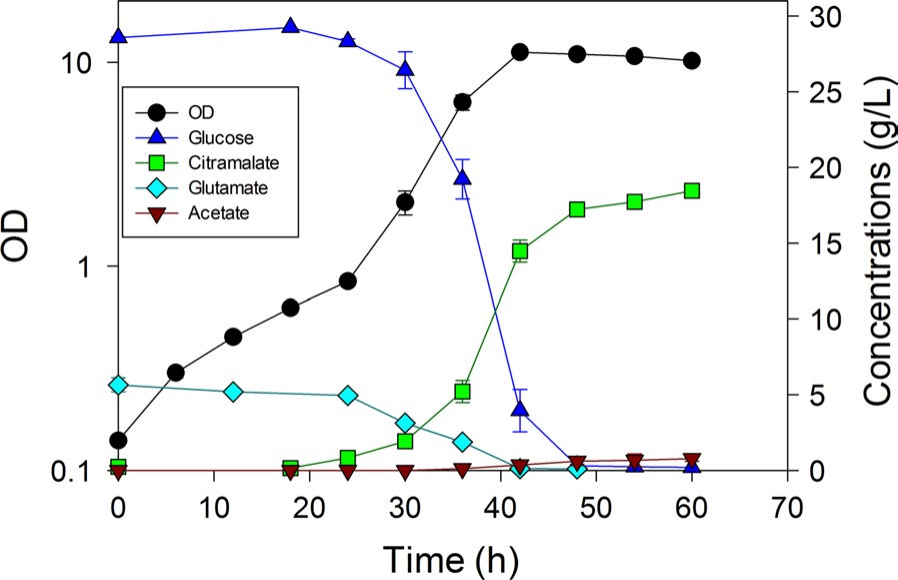
Time course of citramalate production by *E. coli* MEC562/pZE12-*cimA* (∆*gltA* ∆*leuC* ∆*ackA*-*pta*) in duplicate batch culture. The defined medium contained 30 g/L glucose, 5 g/L l-glutamate and 1.0 g/L l-leucine

**Fig. 4:**
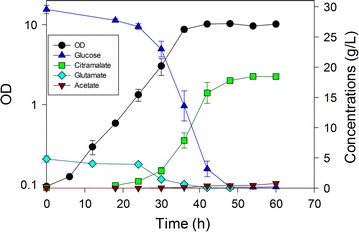
Time course of citramalate production by *E. coli* MEC568/pZE12-*cimA* (∆*gltA* ∆*leuC* ∆*ackA*-*pta* ∆*poxB*) in duplicate batch culture. The defined medium contained 30 g/L glucose, 5 g/L l-glutamate and 1.0 g/L l-leucine

**Fig. 5:**
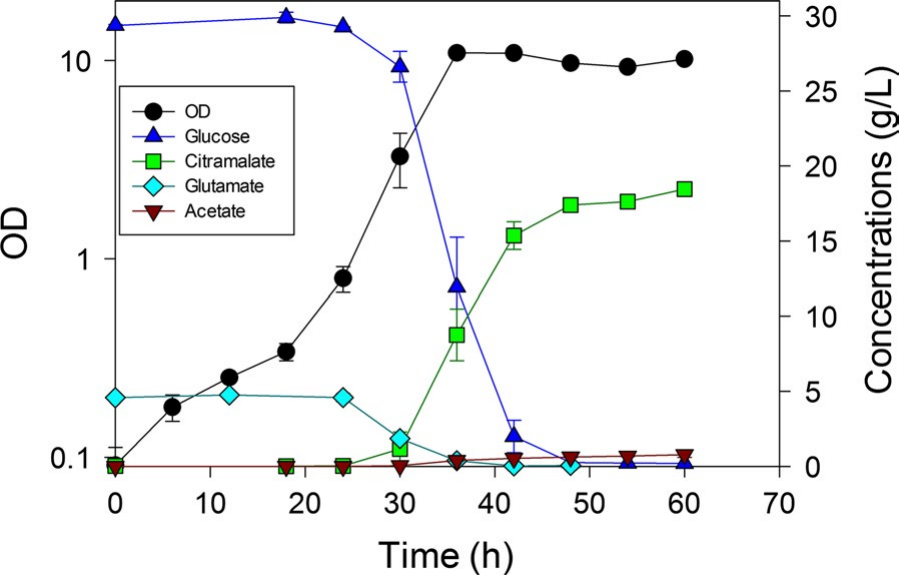
Time course of citramalate production by *E. coli* MEC606/pZE12-*cimA* (∆*gltA* ∆*leuC* ∆*ackA*-*pta* ∆*poxB* ∆*ppsA*) in duplicate batch culture. The defined medium contained 30 g/L glucose, 5 g/L l-glutamate and 1.0 g/L l-leucine

In controlled batch experiments using nominally 30 g/L glucose, we compared these five strains for citramalate yield, acetate yield and volumetric productivity of citramalate (Table 1). As a “control strain”, MEC499/pZE12-*cimA* reached an OD of 13 in 72 h, and accumulated 19.1 g/L (±0.2) citramalate and 1.0 g/L (±0.2). This strain generated citramalate at the slowest rate of all the strains examined. MEC596/pZE12-*cimA* and MEC606/pZE12-*cimA* showed modestly greater citramalate yield than MEC499/pZE12-*cimA* (p = 0.07). MEC596/pZE12-*cimA*, lacking *poxB* but retaining *pta*, showed the lowest acetate yield, but this result did not correspond with the greatest citramalate yield. The highest (statistically indistinguishable) citramalate yields were attained by MEC562/pZE12-*cimA* (Fig. 3) and MEC568/pZE12-*cimA* (Fig. 4) while MEC568/pZE12-*cimA* resulted in lower acetate yield than all other strains except MEC596/pZE12-*cimA*. MEC568/pZE12-*cimA* also showed significantly higher citramalate productivity than all other strains, demonstrating that low acetate formation is accompanied by faster acetyl CoA conversion to citramalate. Despite the fact that the acetate yield was significantly lower using MEC568/pZE12-*cimA* (0.015 g/g) compared to either MEC562/pZE12-*cimA* or MEC606/pZE12-*cimA* (Fig. 5), the citramalate yield from glucose for each of these three strains was similar at 0.60–0.64 g/g. Other potential by-products including succinate, lactate, ethanol, and pyruvate were not detected (<0.02 g/L). The citramalate synthase activity was 31–40 IU/g DCW during the exponential phase for each of the strains.

**Table 1:** Batch fermentations with strains of *E. coli* having knockouts in genes associated with acetate formation and expressing citramalate synthase via the pZE12-*cimA* plasmid

Strain	Key gene deletions	Citramalate yield (g/g)	Acetate yield (g/g)	Volumetric productivity (g/L h)
MEC499/pZE12-*cimA*	*gltA leuC ackA*	0.585^a^	0.030^a^	0.27^a^
MEC562/pZE12-*cimA*	*gltA leuC ackA*-*pta*	0.642^b^	0.027^a^	0.31^b^
MEC596/pZE12-*cimA*	*gltA leuC ackA poxB*	0.609^a^	0.007^c^	0.33^c^
MEC568/pZE12-*cimA*	*gltA leuC ackA*-*pta poxB*	0.626^a,b^	0.015^b^	0.34^d^
MEC606/pZE12-*cimA*	*gltA leuC ackA*-*pta poxB ppsA*	0.604^a^	0.023^a^	0.33^c^

The media for all experiments contained initially 30 g/L glucose, 5 g/L glutamate and 1 g/L leucine. Values in a column with different letters indicate significant difference at p < 0.05

**Fig. 6:**
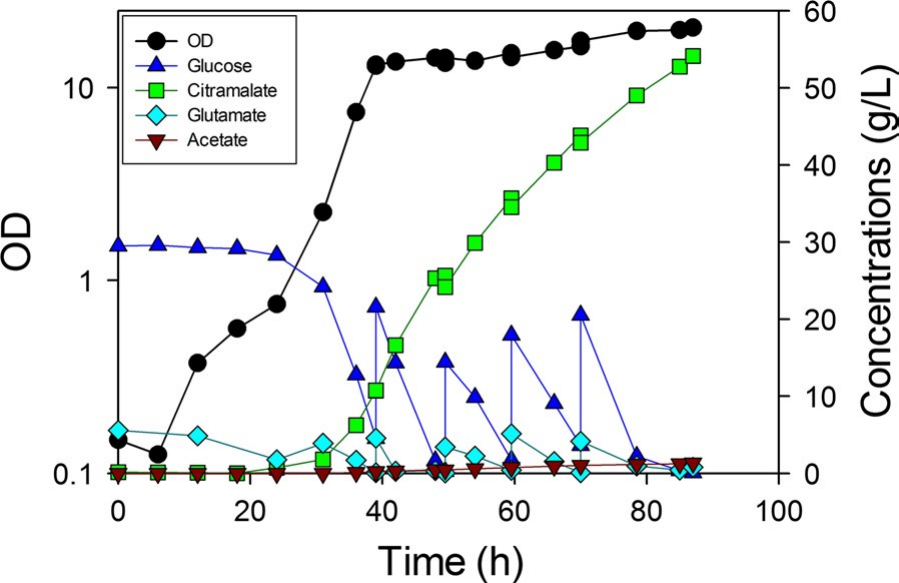
Time course of citramalate production by *E. coli* MEC568/pZE12-*cimA* (∆*gltA* ∆*leuC* ∆*ackA*-*pta* ∆*poxB*) in fed-batch culture. The defined medium initially contained 30 g/L glucose, 5 g/L l-glutamate and 1.0 g/L l-leucine. When the glucose concentration decreased below 5 g/L, 20.0 g glucose, 5.0 g l-glutamate and 1.0 g l-leucine dissolved together in 35 mL DI water were added four times

Strains containing the *poxB* gene deletion (MEC596/pZE12-*cimA* and MEC568/pZE12-*cimA*) generated the least acetate in batch experiments, while the additional *ppsA* knockout (MEC606/pZE12-*cimA*) actually increased acetate formation. Because MEC568/pZE12-*cimA* also had the highest rate of citramalate generation, we chose this strain for a repetitive fed-batch process. Specifically, the process commenced as a batch process, and the glucose concentration was monitored. When the glucose concentration decreased to below 5 g/L, an additional 20 g glucose, 5 g l-glutamate and 1 g l-leucine were added. This batch-wise nutrient feed was accomplished four times during the course of the study, and the OD achieved by the cells after 87 h was 20.5 (Fig. 6). At this time the citramalate concentration was 54.1 g/L, and the yield on glucose was 0.64 g/g, while the acetate concentration was only 1.4 g/L (0.016 g/g yield). Citramalate synthase activity decreased from 35 IU/g DCW at 39 h to 12 IU/g DCW at 87 h. The final mass ratio of citramalate to acetate was approximately 39, and the overall citramalate productivity was 0.62 g/L h, both the highest reported to date.

## Discussion

In this study, citramalate at a high final concentration (54.1 g/L) and yield (0.64 g/g) was formed in an *E. coli* cell factory overexpressing citramalate synthase (*cimA*) gene. We observed over 85% less acetate and a greater citramalate yield compared to a previous study [40]. This reduction in acetate accumulation was accomplished by knocking out the *ackA*-*pta* and *poxB* genes, coding for the two major acetate production pathways in *E. coli*. Some acetate (less than 2 g/L) was still observed during batch and fed-batch fermentation processes in the strains containing *ackA*-*pta* and *poxB* gene deletions (MEC568/pZE12-*cimA* and MEC606/pZE12-*cimA*). In a previous study, only 1.7 g/L acetate from 40 g/L glucose was similarly reported in an *ackA*-*pta* and *poxB* triple mutant *E. coli* strain [31]. In all cases, most acetate accumulation in these triple knockouts occurred in the late exponential and stationary phases. This observation suggests that acetate formation is activated in *ackA*-*pta poxB* strains only when cells are under stress during late exponential and stationary phases, perhaps when a portion of the cellular components are being degraded. Although *poxB* is regulated by the sigma factor encoded by *rpoS* [10] and is preferentially used at low growth rates [1], it is unclear how acetate forms in this triple knockout. Moreover, previous studies reporting acetate formation in *ackA*-*pta poxB* strains, in contrast to the present study, used a complex (LB) medium with glucose [15, 31]. Our study using defined medium suggests that acetate is indeed formed from glucose rather than from an unknown component in a complex medium. Many catabolic reactions generate acetate, and some anabolic pathways including *N*-acetylglucosamine-6-phosphate deacetylase (*nagA* gene), UDP-3-*O*-acyl-*N*-acetylglucosamine deacetylase (*lpxC*), acetylornithine deacetylase (*argE*), cysteine synthases (*cysM* and *cysK*), and acetoacetyl-CoA transferases (*atoA* and *atoD*) pathways might contribute to acetate accumulation [31].

One strategy proposed to reduce acetate accumulation is the overexpression of the acetate scavenging acetyl-CoA synthetase (*acs*) [26]. This pathway helps accumulate acetyl-CoA and hence could benefit citramalate production. Since the saturation of respiratory capacity and resultant increase in the NADH/NAD+ ratio are also known to drive metabolism towards acetate generation [13, 37], efforts to decrease NADH generation may prove useful. For example, expression of NADH oxidase in an *E. coli arcA* mutant eliminated acetate formation at high growth rates [37, 38]. Nevertheless, the complete elimination of acetate while achieving high yield for another product is a challenging problem because it requires a multigene approach and detailed attention to futile pathways, anaplerotic pathways, precursor levels, coenzyme levels, and redox ratios [13]. A comprehensive understanding of the impact of genetic interventions on the metabolic flux distribution through modeling and flux analysis might help fine tune these efforts.

We speculated that knocking out *ackA*-*pta poxB* might result in a greater intracellular accumulation of pyruvate and acetyl-CoA, the precursors of citramalate and hence improve the yield of this biochemical from glucose, though the yield was indistinguishable from the yield previously reported for MG1655 *gltA leuC ackA* expressing citramalate synthase [40]. Glycolysis and the PP pathway are the two major glucose catabolic pathways in *E. coli*, and NADH accumulation during glycolysis induces acetate formation via pyruvate oxidase [37]. Previously, *poxB* mutants have been observed to increase carbon flux through PP pathway by upregulating glucose 6-phosphate dehydrogenase [25]. In this study, the effort to decrease acetate formation by a knockout in the *poxB* gene may have led to the partial redirection of glucose into the PP pathway instead of glycolysis. If such a redirection occurred, any potential improvement in citramalate yield through increased availability of acetyl CoA might be compensated by the loss in yield resulting from the elevated PP pathway flux.

The PP pathway protects cells against oxidative stress by generating reducing equivalents as NADPH [20]. *E. coli* strains blocked in the PP pathway, for example, by deleting the *zwf* gene, compensate for the loss in NADPH formation by increasing glucose uptake rate, increasing the activity of isocitrate dehydrogenase and increasing the TCA cycle flux [29, 43]. Thus, a *zwf* knockout may improve yields of products whose biosynthetic pathways involve glycolysis or TCA cycle metabolites. For instance, *zwf* gene deletion enhanced lycopene production by over 130% in recombinant *E. coli* strains, owing to an improved Emden–Meyerhof–Parnas (EMP) pathway flux and increased pyruvate [44]. Similarly, a 4.4-fold increase in yield was reported in an *E. coli tpiA zwf* strain generating 3-hydroxypropionic acid from glycerol [35]. In our shake flask studies, strains with *zwf* deletions resulted in much lower growth and citramalate accumulation. Unlike previous studies using *zwf* strains, though, the strains in this study also contained a *gltA* knockout that prevented carbon flow from acetyl CoA into the TCA cycle, and glutamate was supplied as a secondary carbon source. Thus, the cells were unable to respond to a block in the PP pathway by generating NADPH in the TCA cycle (i.e., isocitrate dehydrogenase), resulting in significantly reduced glucose uptake and growth rate. Growth and citramalate production in a *zwf* knockout might be improved by engineering another strategy to generate NADPH [27].

We speculated that any intracellular pyruvate accumulation in the *gltA* strain might result in loss of carbon through PEP synthase, and preventing this loss by knocking out the *ppsA* gene could result in pyruvate accumulation. However, no significant benefit of a *ppsA* knockout on citramalate production was observed in the shake flask or batch reactor studies. Gluconeogenic genes are activated in *E. coli* during the metabolic switch from glucose to acetate consumption [23]. Since the strains examined in this study exhausted glucose only at the end of the process and generated low concentrations of acetate, such a switch may not have been a factor, making *ppsA* irrelevant.

ATPase plays a major role in metabolic control, and mutations in ATP synthase increase glycolytic flux [24]. Increased glycolytic flux normally leads to increased acetate excretion through acetate kinase as a means to replenish ATP through substrate level phosphorylation [30]. Growth rate and growth yield are related to the rate of ATP synthesis and the amount of ATP synthesized per unit of substrate consumed [21]. In this study, the strain with the *atpFH* knockouts (MEC638) was unable to generate acetate as a consequence of the *ackA*-*pta poxB* knockouts, and with little metabolic flexibility, failed to grow in the glucose/glutamate/leucine medium.

Potassium hydroxide (KOH) was used for pH control in the fermentation processes. At the end of the fed-batch process (Fig. 6), the concentration of K^+^ ions estimated from the volume of base added to control the pH was 1.1 mol/L, while the NH_4_^+^ concentration was measured to be 188 mg/L. Previous research has demonstrated that the *E. coli* growth ceases at a K^+^ concentration of 1.1 mol/L [41], so the current process may become limited in citramalate formation as a result of the accumulation of the counter-ion needed for pH control.

## Conclusions

This study reports citramalate production at high yield with low acetate accumulation in metabolically engineered *E. coli* overexpressing citramalate synthase by a codon-optimized *cimA* gene. The key knockouts critical to minimizing acetate formation were identified as *pta*, *ackA* and *poxB*. Knockouts of *zwf* and *atpFH* genes, targeted at improving citramalate production by increasing the glycolytic flux and rate, did not show promising results in shake flask studies. Future work will be aimed at further exploring other metabolic and process engineering strategies to achieve higher titers of citramalate without requiring glutamate in the medium while eliminating acetate.

## Methods

### Strain construction and growth media

Strains used in this study are listed in Table 2. The P1 phage method was used for transducing gene mutations into *E. coli* MG1655 from their respective strains in the KEIO collection [5]. When necessary for additional gene deletions, a strain was cured of kanamycin using the pCP20 plasmid [12]. All constructs were confirmed using PCR. All strains were transformed with pZE12-*cimA* plasmid to express citramalate synthase [40]. Strains were routinely grown at 37 °C using Lysogeny Broth (LB). The composition of defined XC medium was (per L): 13.3 g KH_2_PO_4_, 4.0 g (NH_4_)_2_HPO_4_, 8.4 mg Na_2_(EDTA)·2H_2_O, 1.2 g MgSO_4_·7H_2_O, 4.5 mg thiamine·HCl, 13 mg Zn(CH_3_COO)_2_·2H_2_O, 1.5 mg CuCl_2_·2H_2_O, 15.0 mg MnCl_2_·4H_2_O, 2.5 mg CoCl_2_·6H_2_O, 3.0 mg H_3_BO_3_, 2.5 mg Na_2_MoO_4_·2H_2_O, 100 mg Fe(III) citrate, and 100.0 mg citric acid. Carbon sources were added as detailed below. Additionally, either medium was supplemented with 50.0 mg/L ampicillin and/or 100.0 mg/L kanamycin as appropriate.

**Table 2:** Strains used in this study

Strains	Genotype	References
MEC499	MG1655 ∆*gltA770*::(FRT) ∆*leuC778*::(FRT) ∆*ackA778*::Kan	[40]
MEC562	MG1655 ∆*gltA770*::(FRT) ∆*leuC778*::(FRT) ∆*ackA778*::(FRT) ∆*pta779*::Kan	This study
MEC563	MG1655 ∆*gltA770*::(FRT) ∆*leuC778*::(FRT) ∆*ackA778*-*pta779*::(FRT)	This study
MEC564	MEC563 ∆*ppsA776*::Kan	This study
MEC566	MEC563 ∆*acs*-*763*::Kan	This study
MEC568	MEC563 ∆*poxB772*::Kan	This study
MEC570	MEC563 ∆*zwf777*::Kan	This study
MEC576	MEC563 ∆*zwf777*::(FRT) ∆*poxB772*::Kan	This study
MEC596	MG1655 ∆*gltA770*::(FRT) ∆*leuC778*::(FRT) ∆*ackA778*::(FRT) ∆*poxB772*::Kan	This study
MEC606	MEC563 ∆*poxB772*::(FRT) ∆*ppsA776*::Kan	This study
MEC607	MEC563 ∆*poxB772*::(FRT) ∆*zwf777*::(FRT) ∆*ppsA776*::Kan	This study
MEC638	MEC563 ∆*poxB772*::(FRT) ∆*atpFH*::Kan	This study

### Shake flask and bioreactor studies

For shake flask studies, cells were first grown in 3 mL LB for 12–14 h, and then 0.5 mL transferred to 50 mL XC medium with 5.0 g/L glucose, 1.0 g/L l-glutamate and 0.2 g/L l-leucine in 500 mL shake flasks. Each culture was induced at the time of inoculation with 0.2 mM IPTG. Cultures grew at 37 °C and 250 rpm (19 mm pitch) for 24 h. Shake flask studies were replicated three or more times, and statistical analyses were completed using Student’s t test (two-tailed, equal variance), and p < 0.05 was considered the criterion for significance.

All bioreactor studies were conducted in 2.5 L bioreactors (Bioflo 2000, New Brunswick Scientific Co., New Brunswick, NJ, USA). Cultures were again grown first in 3 mL LB, then 50 mL shake flasks as described above, and which were then used to inoculate 1.0 L XC medium with 30.0 g/L glucose, 5.0 g/L l-glutamate and 1.0 g/L l-leucine. Each culture was induced at the time of inoculation with 0.2 mM IPTG. Agitation was maintained at 400 rpm and air supplemented with pure oxygen if necessary was sparged at 1.0 L/min to maintain the dissolved oxygen above 40% saturation. The pH was controlled at 7.0 using 20% (w/v) KOH, and the temperature was maintained at 37 °C. For a fed-batch process, 20.0 g glucose, 5.0 g l-glutamate and 1.0 g l-leucine dissolved together in 35 mL DI water was added four times when the glucose concentration in the culture decreased below 5.0 g/L.

### Analytical methods

Optical density (OD) at 600 nm was measured using a spectrophotometer (UV-650 spectrophotometer, Beckman Instruments, San Jose, CA, USA). Concentrations of extracellular organic acids were measured using HPLC with Refractive Index detection as described previously [16]. Glutamate concentration was measured using a glutamate assay kit (Sigma-Aldrich Co., St. Louis, MO, USA). Ammonia–nitrogen (NH_4_–N) was determined by the Feed and Environmental Water Lab (University of Georgia, Athens, GA, USA) using the colorimetric EPA method [36].

Cell-free extracts were prepared according to the following procedure: (i) centrifuge sample at 3300×*g* for 10 min at 4 °C; (ii) wash the cell pellet twice with 100 mM Tris·HCl (pH 8.0) at 4 °C; (iii) resuspend in 100 mM Tris·HCl (pH 8.0) at 4 °C; (iv) lyse cells using a French^®^ press (Thermospectronic, Rochester, NY, USA) at 14,000 psi with 2–3 passes; (v) remove cell debris by centrifugation at 20,000×*g* for 15 min at 4 °C. Citramalate synthase enzyme activity was measured in the cell-free extracts following a previous protocol [19]. Briefly, the rate of free CoA generated at 37 °C was determined by detecting its reaction product with 5,5′-dithiobis(2-nitrobenzoic acid) at 412 nm.
